# Donor Lungs’ Procurement Implementation with Ex Vivo Lung Perfusion in a Low-Volume Lung Transplant Center

**DOI:** 10.3390/life15010037

**Published:** 2024-12-31

**Authors:** Matteo Petroncini, Elena Salvaterra, Leonardo Valentini, Silvia Bonucchi, Niccolò Daddi, Saverio Pastore, Pietro Bertoglio, Piergiorgio Solli, Filippo Antonacci

**Affiliations:** 1 Division of Thoracic Surgery, IRCCS Azienda Ospedaliero Universitaria Di Bologna, Via Albertoni 15, 40138 Bologna, Italyfilippo.antonacci@aosp.bo.it (F.A.); 2 Division of Interventional Pulmonology, IRCCS Azienda Ospedaliero Universitaria Di Bologna, Via Albertoni 15, 40138 Bologna, Italy; 3 Division of Anesthesiology, IRCCS Azienda Ospedaliero Universitaria Di Bologna, Via Albertoni 15, 40138 Bologna, Italy

**Keywords:** lung transplant, EVLP, lung procurement

## Abstract

(1) Background: Ex Vivo Lung Perfusion (EVLP) is a technique designed to assess and recondition marginal lungs, potentially expanding the donor pool and improving transplant outcomes (2) Methods: This retrospective study evaluated lung transplantation outcomes after EVLP. Donor lungs were assessed using the Toronto protocol, with data on hemodynamics, gas exchange, and perfusion parameters collected and analyzed. Post-transplant complications and survival rates were also examined. (3) Results: Over five years, 17 EVLP procedures were performed. Despite an improvement in lung function, 47% of donor lungs were rejected after EVLP. EVLP-reconditioned lungs showed comparable survival rates to standard transplants, but complications like sepsis and primary graft dysfunction (PGD) occurred. (4) Conclusions: EVLP shows promise in expanding the donor organ availability and reducing PGD, but nearly half of the lungs assessed were rejected. Further research is necessary to optimize EVLP and address potential complications like lung injury and sepsis.

## 1. Introduction

A lung transplant represents the final treatment option for individuals with end-stage lung disease, including conditions such as pulmonary fibrosis, pulmonary hypertension, and COPD. Since the inaugural lung transplantation was conducted in 1963, the procedure has experienced a gradual and sustained global increase in the number of cases performed annually [1]. Nevertheless, the number of lung transplants remains comparatively low in comparison to other organs such as the kidneys, liver, and heart [2]. The scarcity of donor organs represents a significant challenge across the spectrum of transplantation. However, this is particularly evident in the context of lung transplantation, given the inherent limitations associated with this organ. It is estimated that the implantation rate reaches 15–20% of the donations [3]. This can be attributed to the significant fragility of lung tissue, which frequently results in rejection due to early deterioration. Secondly, the discrepancies in standards across Western countries may contribute to a reduction in the number of donations after brain death (DBD). Currently, the assessment of donor lungs involves a series of evaluations, including thorax high-resolution computed tomography (HRCT), blood gas analysis, and bronchoscopy [4]. These examinations are of great importance in reducing the risk of primary graft dysfunction (PGD). PGD represents a significant and unfortunate complication that can arise in the early post-transplant period. It is thought to result from the oxidation damage caused by reperfusion [5].

Traditionally, donor lungs are preserved at low temperatures with the aim of reducing ischemia. However, prolonged ice storage (more than eight hours) may increase the risk of reperfusion damage and, consequently, of PGD [6]. Since the beginning of the 21st century, ex vivo lung perfusion has been employed as a means of addressing the shortage of lung donors, enhancing preservation outcomes and facilitating a novel form of evaluation [7]. It would be remiss not to mention that Cesar Julien Jean Le Gallois is regarded as the father of the conceptualization of an extra-corporeal circulation. In 1895, Jacobi devised an artificial circulation system in which blood was circulated through artificially ventilated lungs. The accidental discovery of heparin by J. McClean, a second-year student at Johns Hopkins in 1915, led to a significant increase in the number of inventions in this field. In 1937, Carrell and Lindberg conducted a single-organ perfusion study, demonstrating organ viability for a period of between 3 and 21 days. The first clinical experience was reported in 2011 by Cypel et al., presenting data about the EVLP application pushing the boundaries of the standard protocols for lung transplant [8]. In 2015, two randomized trials were published, thereby initiating the global dissemination of this technique. This normothermic perfusion after cold static conservation permits the reconditioning of marginal lungs and the utilization of donations after circulatory death (DCD). The Toronto EVLP system necessitates the cannulation of the common pulmonary artery and left atrium, through which the perfusate may be heated to a temperature of 37 °C. At 32 °C, ventilation is initiated via a tracheal cannula at a rate of 7 mL/kg, with seven breaths per minute [7]. Furthermore, EVLP is employed for the reconditioning of standard criteria donors, with the objective of reducing PGD. EVLP enables the assessment and transplantation of high-risk donor lungs with postoperative outcomes that are comparable to those of non-reconditioned lungs in the early postoperative period [9].

While in high-volume transplant centers, EVLP may act as a gatekeeper, allowing time for organ evaluation and the organization of the surgical team. This role is less prominent in low-volume centers, where the primary challenge is donor shortage. Nevertheless, the potential deleterious impact of EVLP remains to be elucidated, necessitating further investigation. The following case series presents a detailed account of our center’s experience with EVLP.2.

## 2. Materials and Methods

All patients provided informed consent. We retrospectively evaluated the outcomes of lung transplantation following Ex Vivo Lung Perfusion (EVLP) since the initiation of our transplantation program. This study included donors with a PaO_2_ of less than 350 mmHg at 100% FiO_2_ and 5 cm H_2_O of PEEP, as well as donation after circulatory death (DCD) donors.

At our institution, the standard donor evaluation protocol includes high-resolution computed tomography (HRCT), bronchoscopy, and arterial blood gas analysis, followed by sternotomy and macroscopic assessment. In the absence of contraindications, a repeat arterial blood gas analysis is performed after 10 min at 100% FiO_2_ and 5 cm H_2_O of PEEP. If the donor lungs are deemed acceptable, the team proceeds with pulmonary artery cannulation, perfusion with 4 °C Perfadex^®^ (XVIVO Perfusion AB, Gothenburg, Sweden) solution, and lung procurement.

Following retrieval, donor lungs undergo retrograde perfusion with 4 °C Perfadex solution and are preserved on ice until implantation or preparation for EVLP.

DCD procedures were conducted in accordance with national guidelines. In Italy, confirmation of death after cardiac arrest requires a mandatory observation period. Specifically, Italian law mandates a 20 min standby period of asystole or absence of vital signs before death can be officially declared. Continuous monitoring, including electrocardiography, is performed during this time to document the absence of electrical and cardiac activity. Once the observation period concludes and all signs of life are confirmed absent, death is officially declared after a final clinical assessment. Subsequently, femoral artero-venous cannulas are placed, and extracorporeal circulation (ECC) is initiated. For DCD involving lungs and liver, a balloon catheter is used to occlude the thoracic aorta.

The procurement process differs depending on whether the heart is included. For heart retrieval, a rapid sternotomy is performed to clamp the supra-aortic trunks and enable central cannulation and transition to ECC, and when peripheral ECC is maintained, left atrial drainage is performed with a cannula positioned in the left atrial appendage. Heart evaluation is then performed, and procurement proceeds as per standard protocols. In lung and liver retrieval without heart procurement, the lungs are rapidly perfused and cooled immediately after ECC initiation [7].

All procedures adhered to the Toronto EVLP protocol [8]. After an additional retrograde perfusion with 4 °C Perfadex, donor lungs were placed in the EVLP dome and connected to a standard cardiopulmonary machine for blood decarboxylation. Ventilation was managed using a Dräger ventilator.

The lungs were connected to the perfusion machine via atrial anastomosis and arterial cannulation. A tracheal tube was attached to the donor trachea, and the lungs were perfused without ventilation for 20–30 min. When the graft reached the temperature of 33 °C, ventilation was initiated.

The EVLP procedure utilized Steen solution, heparin, solumedrol, and antibiotics. A multidisciplinary team, including a surgeon, anesthetist, and perfusionist, conducted a comprehensive multiparametric evaluation of the donor lungs.

For hemodynamic management, pulmonary artery pressure (PAP) was maintained at 8–15 mmHg and left atrial pressure (LAP) at 3–5 mmHg, with adjustments made according to the pulmonary circulation’s response. Once the donor lungs were deemed suitable for transplantation following EVLP, the recipient was prepared in the operating theater, and the lungs were cooled to 10 °C, subsequently perfused retrogradely with 4 °C Perfadex solution, and preserved on ice until implantation.

Hemodynamic and respiratory gas exchange data were collected and analyzed using a *t*-test for statistical validation.

## 3. Results

### 3.1. Patients

The general data about EVLP cases are reported in Table 1. The first lung transplant in our center was performed in 2001. Since that time, the number of transplant procedures has increased on a regular basis, with no significant interruptions. A total of 124 lung transplants were performed, 14 of which were conducted in the most recent year. Monolateral lung transplants have been performed in only 15 cases in total. The overall survival rate is approximately 47.1%. In 2019, our center introduced the application of EVLP and the first use was on 22 March 2019 for marginal lung procurement, with a positive effect on the graft. In that situation, lungs were implanted in a patient requiring an urgent transplant for ARDS. Unfortunately, the initial experience yielded unfavorable results, resulting in the patient’s demise three days later due to sepsis and PGD. Since 2019, we have received 1709 offers of lungs, 140 (8.1%) of which were evaluated and 83 (4.8%) have been accepted.

Over a five-year period, 15 EVLP procedures were conducted, in 8 instances occurring subsequent to DCD (2 of which were combined heart, lung, and liver DCD). The remaining procedures were performed due to a PaO_2_ below 350 mmHg during the evaluation of the donor lungs. Over the past 18 months, there has been a notable enhancement in the utilization of EVLP, with approximately half of the total cases conducted during this period (eight cases). A total of eight cases, representing 47% of the overall cases, were implanted after a minimum of 100 min of perfusion. The mean duration of extra-corporeal lung perfusion (EVLP) is 222 min, with a maximum of 295 min. The majority of donor lung procurements were conducted at regional centers, thereby obviating the necessity for lengthy travel. Two cases were transported from an external facility, situated 450 km and 1300 km away, respectively, from our institution (both by airplane). A dearth of data pertaining to the initial 15 cases was observed due to the utilization of the home-assembled EVLP apparatus necessitating a division between ventilation and perfusion. It is, therefore, not possible to comment on any potential deterioration in pulmonary compliance.

The macroscopic evaluation represents a crucial point of assessment. Some features of the lung parenchyma, such as weight, elasticity, and the presence of foamy secretions, can be observed visually and palpated. Despite the favorable macroscopic evaluation, four cases of donor lung rejection occurred due to the presence of unacceptable functional features. Conversely, in three cases, a decline in the oxygen exchange led to implantation in the context of a favorable palpatory lung presentation. It is possible for the macroscopic degradation of the lung parenchyma to occur rapidly and between near evaluations. Furthermore, secretions monitoring should be conducted, with particular attention paid to the production of foam by bronchial tree, as this is indicative of significant edema.

### 3.2. Perfusion

Table 2 presents the findings with regard to the perfusion values, divided per hour of ex vivo lung perfusion (EVLP). In the event of a discernible decline in the condition of the donor organs, the perfusion was terminated prior to the four-hour evaluation. The mean flow rate throughout the entire EVLP period was 1.89 L/min, exhibiting minimal fluctuations at different time points. Additionally, pressure values were recorded in the pulmonary artery (PAP) and the left ventricle (LVP), with a mean value of 10.33 mmHg and 4.42 mmHg, respectively. Vascular pulmonary resistances (VPR) were found to be 5.31. Finally, pO_2_ and pCO_2_ were evaluated before and after the EVLP machine. The mean value of pO_2_ before the machine was 286.87 mmHg and pCO_2_ was 28.08 mmHg.

### 3.3. Accepted vs. Rejected Donor Lungs

Table 2 presents a comparison between the accepted and rejected group data. The results obtained were intriguing, although the statistical significance was low due to the limited number of cases examined. As anticipated, there were no significant variations in the flow rate over time or between the two groups. A contrasting scenario is illustrated by PAP, which demonstrates a consistent upward trend in the rejected group. The LAP analysis yielded the sole statistically significant result, namely that the rejected donor lungs exhibited higher values (up to 5.0 mmHg vs. 3.7 mmHg mean value). A similar trend is evident in the PVR data, whereby vascular resistance increases with each passing hour. The final and most crucial aspect of the lung function analysis is the assessment of gas exchange efficiency. The PaO_2_ was evaluated both before and after oxygenator. An increase in PaO_2_ prior to the oxygenation, was observed in accepted lungs. The initial value was recorded at 245.5 mmHg, increasing to a mean of 293.0 mmHg by the fourth hour. It was unexpected that the values observed in the rejected lungs were higher (311.7 mmHg at the first hour to 359.0 mmHg at the fourth hour). Conversely, PaCO_2_ demonstrated a rapid trajectory towards elevated values in the accepted lungs (22.5–37.0 mmHg), while the rejected lungs exhibited stable mean values. The final aspect of our evaluation pertained to the replacement of the solution and the duration of the procedure for lungs that were accepted and those that were rejected. Steen solution is replaced at regular intervals as indicated in the Toronto protocol or in case of fluid sequestration by the lungs, and this finding was considered as a sign of lung dysfunction. At the first and fourth hours, a greater mean volume of replaced solution was observed in the accepted group, whereas the opposite was noted at the second and third hours. The mean duration of the EVLP procedure for the lungs that was accepted was approximately 249 min, while the mean duration for the rejected lungs was 190 min.

### 3.4. Post-Operative Results

In Table 3, we reported the postoperative results. Furthermore, the perioperative outcomes were collected for the eight patients who underwent transplantation with EVLP-reconditioned lungs. In each case, the procedure was supported by intraoperative ECMO, which was prolonged prophylactically for a minimum of two days and a maximum of six days. The mean duration of mechanical ventilation was observed to be high (144 h), with only two patients not requiring a tracheostomy. No cases of a redo for bleeding were observed; however, two patients were taken to the operating theatre for non-pulmonary reasons (one case of ECMO cannula repositioning and one lymphocele revision). Two patients developed grade 3 PGD at 72 h. The first application of EVLP (Case 1) resulted in an intraoperative death due to uncontrolled bleeding, making the postoperative evaluation of our first experience impossible. A total of three in-hospital deaths were recorded, two attributable to sepsis and multiorgan failure and the other one to intraoperative issues. Following discharge, only one case of late-onset chronic rejection was observed, resulting in the patient’s demise. We did not find any significant difference between the EVLP cohort and our general transplant population in terms of the postoperative outcomes.

## 4. Discussion

EVLP represents a relatively novel approach that is currently undergoing a process of scientific evaluation. To date, the highest level of evidence is represented by a small number of randomized trials exploring the potential advantages of this technique in large-volume centers. The objective of this study is to assess the potential benefits of EVLP in low-volume-center procurement. Over the years, there has been a notable surge in the utilization of EVLP, with the exception of the period marked by the global pandemic caused by the SARS-CoV-2 virus. Since the initial implementation of EVLP at our institution in 2019, we have employed normothermic perfusion to assess lung viability in 0.8% of the total organ offers. Our experience suggests that EVLP enables a substantial number of implantations that would otherwise not have been performed if the lungs had been evaluated solely during the procurement process. This finding lends further support to the validity of the decision-making process in relation to organ procurement. It is important to note, however, that in these cases, a significant proportion of the EVLP procedure was conducted following DCD donations, rather than for marginal features of the lungs. In these cases, it would be more beneficial to assess the preservation and reconditioning capability.

During the initial implementation of EVLP in clinical practice, several fundamental aspects were identified and discussed. Indeed, the initial application of this technology was intended for the evaluation and reduction of the ischemic time in the conservation of donor lungs. However, it became evident that the potential for application was multi-faceted, and several centers investigated the use of EVLP as a means of improving organ function and drug administration.

EVLP is capable of maintaining stable conditions for a period of 12 h without a notable decline in respiratory performance [7]. This assumption alters the projected outcome, transforming EVLP from a salvage strategy prior to rejection into a viable option for standard lungs.

One of the earliest randomized trials to investigate the EVLP procedure was conducted at Toronto General Hospital by Cypel et al. [8]. Over a two-year period, they collected data on 23 EVLP cases, with only three lungs (13%) being rejected. Despite a similar distribution between DBD and DCD donations, a higher ratio of rejected lungs was observed after EVLP (47.0%), which may be attributed to the selection process. It is notable that, although the EVLP preservation was applied to 20.7% of the overall harvested lungs in the Cypel cohort, compared to 28.3% in our study, the total number of rejected lungs is lower than our rejecting ratio (55.6% vs. 95.2%). This may be attributed to a heightened awareness of lung preservation techniques prior to harvesting and a more assured approach to procurement, particularly given that the minimum pO_2_ value for lungs implanted without EVLP was 290 mmHg. This underscores a growing conviction that the macroscopic evaluation of the organ plays a pivotal role in lung assessment.

A notable discrepancy was observed between the DCD and DBD donors with respect to pO_2_ levels within the donor. The trial observed higher values of pO_2_ in DCD lungs compared to donor lungs. This finding is not unexpected, given that in our countries, EVLP is a standard practice following DCD, even when the lung performance is suboptimal. The only significant perioperative data provided by Cypel in his perioperative results are related to PGD at 24 h, which he found to be significantly higher in the non-EVLP cohort (with a difference of 21 percentage points). In our experience, we only evaluated PGD grade 3 at 72 h, and we did not encounter any cases of this complication. Furthermore, a significant finding of the aforementioned trial is the rejection rate, with approximately 13% (3 cases) of the EVLP cohort not implanted due to a decline in lung function. Conversely, a greater proportion of lungs (47%) were rejected following EVLP in our series, with a notable decline in the respiratory capability. The identification of the mechanism responsible for this loss of function in donor organs may prove challenging. It is not possible to establish a causal relationship between the detrimental process and either intrinsic lung dysfunction or perfusion.

Similarly, in 2017, Slama et al. [10] presented the findings of the inaugural randomized trial on the EVLP experience. In essence, the researchers compared the outcomes of standard organ preservation methods with and without the use of EVLP. They demonstrated that the postoperative outcomes of organs preserved using EVLP are comparable to those achieved through conventional methods [10]. This evidence expanded the potential for EVLP application to all donor lungs, even those with a standard function, thereby facilitating the procurement process. Nevertheless, these two pieces of high-level scientific evidence are based on the assumption that EVLP could only provide an improvement in the donor lung function. However, there is currently no evidence that excludes the possibility of EVLP-related tissue damage.

Although, as it has been previously demonstrated, EVLP is a safe procedure for lung conservation and a useful method for prolonging the evaluation period, and evidence regarding its reconditioning capabilities is more limited. The majority of the cases included in the initial cohort were lungs that had been initially rejected, with only a few cases following DCD. It is not uncommon for a greater number of rejected lungs to be identified during the initial stages of implementing this technique. Consequently, in 2012, Aigner et al. determined that approximately 31% of donor lungs subjected to EVLP were unsuitable [11]. Conversely, a French study comprising 32 EVLP cases demonstrated that 31 (96, 8%) exhibited a notable improvement, resulting in subsequent implantation [12].

Furthermore, a review conducted by Braithwaite et al. in 2023 postulated the potential for ventilation injuries to occur during EVLP [13]. It is possible that predictable, unadjusted pressure and volume values may generate mechanical tension against the alveolar tissues. Conversely, the release of the ventilation pressure and pronation appear to exert a beneficial influence on lung function. In our cases, we encountered a compatible scenario. Indeed, in several cases, we observed a high PaO_2_ value initially, which then declined significantly during the evaluation period. It is unlikely that this deterioration can be attributed to an intrinsic impairment of the donor lungs.

Moreover, Valenza and colleagues conducted an evaluation of 39 donor lungs between 2011 and 2013 [14]. The randomized trial presented features comparable to our experience, due to the geographical affiliation. Indeed, three lungs that did not meet the acceptance criteria were rejected without consideration of the EVLP option. Conversely, EVLP was employed in only eight cases, in seven instances due to a PaO_2_ under 300 mmHg and in one instance for other reasons. A single rejection was observed, along with seven improvements following EVLP. It is noteworthy that in the EVLP group, a more pronounced reduction in PaO_2_ was observed between the optimal measurement during DCD certification and the immediate pre-harvesting period. It seems reasonable to posit that a prolonged period of time spent in the donor organism following DCD could have a significant impact on the lungs, which may be fully recovered by the EVLP.

Ultimately, the postoperative data align with the broader transplant series. As reported by Wallinder et al. [15], PGD is more frequent in the EVLP-reconditioned organ recipient; however, we did not find any case of PGD grade III at 72 h. Conversely, we identified 15 (12, 1%) cases of PGD grade 3 at 72 h in patients transplanted without an EVLP procedure. Otherwise, Wallinder also reported a mean length of stay of 4 days. Differently, in our EVLP cohort, we saw a much longer ICU stay (mean value 14 days), maybe due to the inclusion of 72 h of prophylactic ECMO after every transplantation in our protocol. The overall length of stay in our EVLP cohort was shorter than the mean of our entire series, although we hypothesize that this finding may be related to the inclusion of our early cases [15]. The overall survival rate was 62.5%, which is higher than the overall survival rate of standard transplantation. Indeed, Valenza et al. observed no statistically significant difference between reconstructed EVLP and standard transplantation in their study [15].

However, given the small cohort size, it is challenging to draw meaningful comparisons between this EVLP group and patients who did not undergo EVLP.

The suitability of small lung transplant centers to provide effective lung transplantation depends on their ability to maintain high standards of care, multidisciplinary expertise, and a sufficient volume to ensure competence and optimal outcomes. Smaller centers may offer advantages such as personalized care, shorter waiting times, and localized access for patients in geographically underserved areas. However, challenges such as limited resources, difficulties in maintaining robust training programs, and potential variability in outcomes due to lower case volumes require rigorous adherence to national and international guidelines, robust outcome monitoring, and integration into larger transplant networks to optimize care and equity for all patients.

## 5. Conclusions

The scarcity and fragility of donor lungs impose significant limitations on the number of transplants that can be performed, necessitating the development of innovative approaches such as EVLP. Our experience with EVLP demonstrates its potential to expand the donor pool and improve the viability of marginal lungs, particularly in low-volume centers. Although our data indicate that EVLP can improve lung function and reduce the incidence of PGD, they also underscore the intricacies of the donor lung assessment and the variability in outcomes. Despite the encouraging outcomes, nearly half of the lungs assessed with EVLP were ultimately rejected, underscoring the necessity for enhanced expertise in this application to fully realize the potential advantages of this procedure. Furthermore, the survival rate of recipients of EVLP-reconditioned lungs was comparable to, or even superior to, that of traditional transplant methods, indicating that EVLP can be a valuable tool in lung transplantation. Nevertheless, the potential for complications such as sepsis and the possibility of EVLP-related lung injury necessitate further investigation. In conclusion, while ex vivo lung perfusion (EVLP) represents a significant advancement in lung transplantation, further research is required to optimize its use and fully understand its long-term impact on patient outcomes.

## Figures and Tables

**Table 1:** Preoperative data.

EVLP	Year	Indication	HLL/LL DCD	Implantation	EVLP Time (min)	Recipient Diagnosis
Case 1	2011	pO_2_ < 350 mmHg	-	Yes	229	IPF
Case 2	2019	DCD	LL	Yes	240	PAH
Case 3	2019	pO_2_ < 350 mmHg	-	Yes	237	PAH
Case 4	2019	pO_2_ < 350 mmHg	-	Yes	282	IPF
Case 5	2020	DCD	LL	No	210	BE
Case 6	2021	pO_2_ < 350 mmHg	-	No	134	PAH
Case 7	2022	DCD	LL	Yes	255	PAH
Case 8	2022	pO₂ < 350 mmHg	-	No	265	COPD
Case 9	2022	pO_2_ < 350 mmHg	-	Yes	282	IPF
Case 10	2023	pO_2_ < 350 mmHg	LL	No	184	IPF
Case 11	2023	DCD	LL	Yes	264	IPF
Case 12	2023	DCD	HLL	No	295	COPD
Case 13	2023	DCD	HLL	Yes	209	PAH
Case 14	2023	DCD	LL	No	145	PAH
Case 15	2023	DCD	LL	No	100	PAH

BE: Bronchiectasis, COPD: Chronic Obstructive Pneumopathy Disease, HLL: Heart, Lungs, and Liver procurement, IPF: Idiopathic Pulmonary Fibrosis, LL: Lungs and Liver procurement, PAH: Pulmonary Arterial Hypertension.

**Table 2:** Perfusion data of accepted and rejected donor lungs.

	1 h	2 h	3 h	4 h	*p*-Value
Flow rate (L/min)					
Accepted	1.87 (2.1-1.6)	1.93 (2.4-1.6)	1.94 (2.3-1.6)	1.87 (2.4-1.9)	N.S.
Rejected	1.85 (2.0-1.5)	1.87 (2.1-1.5)	1.93 (2.1-1.8)	1.96 (2.1-1.9)	
PAP (mmHg)					
Accepted	9.75 (14-8)	10.13 (14-7)	10.86 (16-7)	9.17 (12-7)	N.S.
Rejected	9.67 (12-8)	11 (15-8)	11 (14-8)	13.5 (19-8)	
LAP (mmHg)					
Accepted	4.8 (7.0-3.0)	4.6 (6.0-3.0)	3.2 (4.0-3.0)	3.6 (5.0-2.0)	N.S.
Rejected	5.5 (8.0-3.0)	5.4 (7.0-4.0)	4.3 (5.0-4.0)	5.1 (7.0-3.0)	
PVR (dyn sec/cm^−5^)					
Accepted	210.34 (400-114.2)	234.56 (365.7-100)	285.54 (489-150.5)	225.64 (320-134)	N.S.
Rejected	209.34 (366.1-125)	262.10 (385-118.8)	276.84 (375-157.6)	351.31 (503-200)	
Arterial pO_2_ (mmHg)					
Accepted	338.81 (588-264)	339.84 (447-231)	377.41 (448-290)	398.38 (404-328)	N.S.
Rejected	351.96 (491-278)	298.27 (359-200)	342.04 (366-306)	359.02 (395-323)	
Arterial pCO_2_ (mmHg)					
Accepted	22.52 (28-16)	23.63 (31-18.2)	29.89 (57.9-20)	36.98 (85-19)	N.S.
Rejected	22.56 (31-16.1)	28.48 (44.5-19.5)	32.33 (56.7-22)	22.45 (24.9-20)	
Venous pO_2_ (mmHg)					
Accepted	338.82 (588-264)	339.87 (447-231)	377.44 (448-290)	398.32 (404-328)	N.S.
Rejected	351.94 (491-278)	298.2 (359-200)	342.09 (366-306)	359.03 (395-323)	
Venous pCO_2_ (mmHg)					
Accepted	28.51 (38-20.2)	30.61 (39-25.1)	31.54 (41-18)	44.83 (121-23)	N.S.
Rejected	28.09 (34-19.8)	37.83 (68-24.1)	32.76 (37-23)	29.65 (32.3-27)	
Solution replacement (mL)					
Accepted	660 (1000-500)	250 (250-250)	285 (500-250)	308 (500-250)	N.S.
Rejected	505 (540-500)	266 (350-250)	250 (250-250)	250 (250-250)	
Duration (min)					
Accepted	249 (282-209)				0.036
Rejected	190 (295-100)				

PAP: Pulmonary Arterial Pressure, LAP: Left Atrial Pressure, N.S.: not significant, PVR: Pulmonary Vascular Resistance.

**Table 3:** Postoperative results.

EVLP	Case 1	Case 2	Case 3	Case 4	Case 7	Case 9	Case 11	Case 13
Prolonged ECMO	NA	Yes	Yes	Yes	Yes	Yes	Yes	Yes
Duration of Prolonged ECMO (d)	NA	3	2	4	5	6	4	6
Mechanical Ventilation (h)	NA	96	72	168	168	168	168	192
Tracheostomy	NA	No	Yes	Yes	No	Yes	Yes	Yes
Redo	NA	Yes	No	No	Yes	No	No	No
Dialysis	NA	No	No	No	Yes	Yes	Yes	Yes
Atrial Fibrillation	NA	No	No	No	No	No	No	No
PGD Grade 3 at 72 h	NA	No	No	Yes	No	Yes	No	No
Stroke	NA	No	No	No	No	No	No	No
Sepsis With MOF	NA	No	No	Yes	No	Yes	No	No

MOF: multi-organ failure, NA: not assessable, PGD: primary graft dysfunction.

## Data Availability

The data presented in this study are available on request from the corresponding author due to privacy and ethical reasons.
